# Caring for the careers: A psychosocial support model for healthcare workers during a pandemic

**DOI:** 10.4102/curationis.v46i1.2430

**Published:** 2023-06-21

**Authors:** Idah Moyo, Livhuwani Tshivhase, Azwihangwisi H. Mavhandu-Mudzusi

**Affiliations:** 1 HIV Services, Population Solution for Health, Harare, Zimbabwe; 2 Department of Health Sciences, College of Human Sciences, University of South Africa, Pretoria, South Africa; 3 Department of Nursing Sciences, School of Health Care Sciences, Sefako Makgatho Health Sciences University, Pretoria, South Africa; 4 Department of Graduate Studies and Research, College of Human Sciences, University of South Africa, Pretoria, South Africa

**Keywords:** caring, healthcare workers, model, pandemic, psychosocial, support

## Abstract

**Background:**

The coronavirus disease 2019 (COVID-19) pandemic has further placed additional stress on the already fragile and overstretched healthcare system in Zimbabwe. Most healthcare institutions reported staff shortages, inability to cope with the extra workload, burnout and the resultant psychological implications.

**Objectives:**

This study aimed to develop a psychosocial support model that sustains a support structure that will contribute to an enabling work environment promoting efficiency and effectiveness in response to public health emergencies.

**Method:**

Empirical findings from interpretive phenomenological analysis studies on healthcare workers’ experiences during the COVID-19 in Zimbabwe formed the basis for model development. The model development in this study was informed by the work of Donabedian, Dickoff, James and Wiedenbach, Walker and Avant, Chinn, Kramer and Wilkes.

**Results:**

The developed model is described using the elements of Donabedian’s framework (structure, process and outcome) and of Dickoff, James and Wiedenbach’s (1968) practice theory elements (agents, recipients, context, process, dynamics and outcome) and within the national and international context of the COVID-19 pandemic.

**Conclusion:**

The fragile and under-resourced healthcare system has psychosocial implications to the well-being of healthcare workers. The utilisation of this model is critical and facilitates the provision of an enabling and supportive environment that facilitates efficiency in response activities during pandemics.

**Contribution:**

This study provides a reference guide in the provision of psychosocial support for healthcare workers particularly during public health emergencies. There is paucity of evidence focusing on the well-being of healthcare workers during a crisis, hence the significance of this study.

## Introduction

Since the outbreak of the coronavirus disease 2019 (COVID-19) reached pandemic status in 2020, the healthcare system has not stabilised in some countries such as Zimbabwe, with the pandemic having caused extreme stress (Nie et al. 2021). The World Health Organization (WHO) had to step in and advised countries to implement lockdowns in order to limit the spread of the new virus, about which little was known initially (WHO 2020). Healthcare providers were among those most severely affected by the COVID-19 pandemic, as they were required to continue rendering healthcare services. In addition, they were the frontline workers, who witnessed at first hand the severity of the disease among the patients admitted into their care.

The COVID-19 pandemic placed additional stress on the already fragile healthcare system in Zimbabwe. Makoni (2020) reports that the healthcare services in that country (Zimbabwe) were overstretched even before the COVID-19 pandemic, but that COVID-19 infections among healthcare workers and the inadequate supply of personal protective equipment (PPE) further worsened the situation. Similar burdens placed on frontline healthcare professionals were also reported in Bangladesh, where an increased workload and a potential system failure in the healthcare sector were experienced during the crisis (Razu et al. 2021).

Although they were aware of the importance of social distancing, healthcare professionals were unable to practice it, as their work required them to be in close proximity to the patients they were caring for. This undoubtedly caused extreme stress, as they witnessed the severity of the virus at close range. They therefore lived with the possibility of themselves becoming infected with the virus and that they might then transmit infection to their next of kin when they left work. The possibility of death as a result of COVID-19 was an ever-present reality, as they witnessed those in their care dying daily in numbers (Moyo et al. 2021; Liang Wu & Wu 2021).

Most healthcare institutions reported staff shortages, with there being insufficient healthcare workers to cope with the extra workload arising from the COVID-19 outbreak. A study conducted in Limpopo province in South Africa revealed severe shortages of healthcare providers, with nurses being required in consequence to work long shifts to cover for those who were ill and in quarantine (Moyo et al. 2022).

De Raeve, Adams and Xyrichis (2021) in a study on the impact of the pandemic on nurses in Europe found the infection, hospitalisation and death of nurses to be causes of distress and reported a lack of uniformity in the compensation offered by the governments of various European countries to those who contracted COVID-19 as a result of their occupation.

Healthcare providers were affected psychologically and experienced burnout, leading many to express their intention to leave the profession, which further exacerbated staff shortages. Nurses were reported as suffering from depression as a result of nursing COVID-19 patients (EFN 2020b cited in De Raeve et al. 2021). Some experienced stigmatisation, discrimination and eviction from rented accommodation, while others experienced verbal abuse. Psychological obstacles such as these were worsened by a lack of social support from employers in many countries (De Raeve et al. 2021; Xu, Stjernswärd & Glasdam 2021). In light of the preceding factors and the indispensable role that frontline healthcare workers fulfil, the provision of psychosocial support for them is crucial.

Following this introduction, the article will outline the purpose, measures of trustworthiness and ethical considerations for the study. The article will further discuss the theoretical foundations of the model and describe how Donabedian approach and Dickoff, James and Wiedenbach’s (1968) practice theory will be applied in the development of the model. The model and its elements will be described within the global, national, and institutional level contexts, followed by highlighting the main outcome and application of the model.

## Application of the Donabedian approach in the development of the model

The Donabedian framework was found appropriate for assessing the environment in which healthcare workers perform their duties so as to ensure the provision of quality care. The framework makes provision for the assessment of structure, process and outcome. According to the Donabedian framework, structure refers to factors that influence the context; in this case these are finances, human resources and material resources such as equipment (Donabedian 1988). In the context of the study conducted, financial resources are necessary for the purchasing of equipment such as PPE, which was essential for ensuring the protection and physical well-being of healthcare professionals during the COVID-19 pandemic. Human resources in this context are inclusive of management and healthcare workers, who are needed in greater numbers so as to ease the workload created by COVID-19. On the other hand, process refers to what is being done, in the sense of the implementation of the psychosocial model of care in the model study under discussion (Botma & Labuschagne 2019; Donabedian 1988). This process relates to the institutional support that ensures that all healthcare providers affected by or infected with COVID-19 receive counselling, training and support. The anticipated outcome is a supportive work environment that enhances the psychological well-being of healthcare providers.

### Purpose

The purpose of the study was to develop and describe a psychosocial model for enhancing psychosocial support for healthcare workers during COVID-19 and other public health emergencies.

### Measures to ensure trustworthiness

According to Moule, Aveyard and Goodman (2016), trustworthiness refers to a method of establishing or ensuring scientific rigour in qualitative research, without sacrificing relevance. The researchers complied with measures aimed at ensuring credibility, dependability, confirmability and transferability. Moule et al. (2016) defined credibility as the degree to which the study’s findings are a true reflection of the experiences and perceptions of the study’s participants. To enhance credibility, the researchers made every effort to build rapport and a relationship of trust with the research participants through prolonged engagement, bracketing and peer debriefing. Transferability was ensured through the use of dense descriptions of the participants’ lived experiences, as well as demographic information and the use of direct quotes. To further enhance trustworthiness, the model was shared and reviewed by nurse managers, nurse educators and nurses who had either suffered from COVID-19 or conducted COVID-19 response activities.

### Design

**FIGURE 1:**
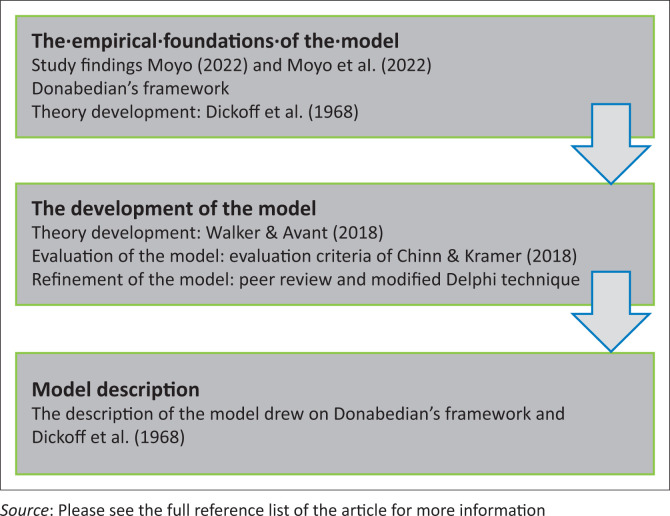
The empirical foundations of the model.

The design of the model took the form of three sequential phases, namely presentation of the empirical foundation of the model, development of the model, and description of the model. Donabedian’s theory (Donabedian 1980a, 1980b) and the work of Dickoff et al. (1968) constituted the theoretical framework guiding the model development process. The model further drew on study’s findings by Moyo (2022) and Moyo et al. (2022). Figure 1 shows the processes followed in developing the model.

### Theory development approaches

Perspectives from divergent frameworks as well as empirical findings were integrated to facilitate theory development, which was informed by the work of Donabedian (1980a, 1980b), Dickoff et al. (1968), Walker and Avant (2018), Chinn, Kramer & Sitzman (2021), Veugelers et al. (2020) and Wilkes (2015). The basis of the concept analysis, synthesis and derivation was provided by the work of Walker and Avant (2018) and Chinn and Kramer (2018). The modified Delphi technique was utilised for evaluation and refinement of the model (Veugelers et al. 2020; Wilkes 2015). The process that was followed in developing the model is illustrated in Figure 1, and a detailed description of the key steps of the process follows.

### The empirical foundations of the model

In order to understand how services can be improved, the researchers found it important to identify how healthcare services were being implemented in the context of COVID-19. Therefore, the model is based on the findings of interpretive phenomenological analysis studies conducted in Zimbabwe by Moyo (2020) and Moyo et al. (2022).

The interpretative phenomenological analysis (IPA) design (Smith & Osborn 2015) was employed to gain insight into the lived experiences of healthcare workers who either provided care to COVID-19 patients or themselves contracted COVID-19. This approach enabled the researcher to gain an in-depth understanding of individual healthcare worker’s experiences during the COVID-19 period. Purposive sampling was utilised to gain access to study participants (Alase 2017). A sample size of 20 participants, aged between 25 and 40 years, was attained following data saturation. Of the healthcare workers who participated in the study, 10 were providers of care, while the other 10 had contracted COVID-19. Data were collected using in-depth interviews that were audio-recorded. Data analysis were performed using the IPA framework. The following steps, as identified by Smith and Osborn (2015), were followed: (1) reading and re-reading the transcript; (2) note taking and developing emergent themes; (3) clustering the emergent themes; (4) crafting a master table of themes composed of superordinate themes, subthemes, and extracts from the interviews; (5) examining and identifying the similarities between the master tables of the themes; and (6) compiling a single master list comprised a superordinate theme, themes, and sub-themes. This resulted in themes and sub-themes describing the experiences of healthcare workers as either providers of care or as patients having contracted COVID-19 (see Box 1, summary of research findings and the outline that follows). The gaps that emerged are associated with the burden created by COVID-19 in an already fragile and overextended healthcare system (Makoni 2020).

BOX 1 Summary of key research findings that formed the basis of the development of the model.

**Table:** 

**Structure**	**A lack of institutional support structure** The study established that the study setting lacked an institutional support structure for healthcare workers who participated in COVID-19 response activities. Nurses who contracted COVID-19 felt unappreciated because no follow-up was conducted to check whether they were recovering or were experiencing any difficulties.
	**Shortage of human resources** Findings showed inadequate staffing levels in the face of an increased workload arising from the response to the COVID-19 pandemic. Staff shortages were prevalent in various sections where care was provided for COVID-19 patients, but the situation was even worse for those in the Rapid Response Team. The team indicated that the workload was overwhelming, strenuous and emotionally draining because of the COVID-19 contact tracing activities that had to be conducted daily. This resulted in the healthcare workers working long shifts of 12 h or more. In addition, the nurses felt that they had been pushed into the battle against COVID-19 without adequate preparation and support. Moreover, the study found participants to be unprepared when they began caring for severely ill COVID-19 patients; this lack of preparedness increased their psychological stress and fear.
	**Inadequate material resources and equipment** The study’s findings established that the healthcare environment where care was provided during the COVID-19 period was lacking in appropriate medical resources and equipment, for example, few hospital departments had piped oxygen when it was most needed. Equipment such as blood pressure and blood sugar testing machines (glucometers and glucostix) were also in short supply, with diabetic patients having to purchase these themselves in some instances. It also emerged that there were insufficient supplies of PPE, particularly during the first wave of COVID-19. This made it difficult for the healthcare workers to provide the appropriate standard of care, and this affected them emotionally, triggering high levels of stress and anxiety, as healthcare workers were worried about their safety and feared cross infection.
	**Healthcare- and treatment-related costs** It emerged from the study that some study participants (healthcare workers) who had contracted COVID-19 experienced out of pocket expenses in the form of healthcare- and treatment-related costs, for example, sick healthcare workers had to pay for X-rays, COVID-19 PCR testing and medicines. In addition to battling with illness, participants also had to come up with the funds to cover medical expenses, which should have been attended to by the hospital at which they were working.
**Processes**	**Communication, training, and support** The study’s findings showed inadequate communication between the nurses involved in COVID-19 activities and their managers. Study participants were not given an opportunity to express their concerns, fears and experiences relating to difficulties they encountered in the execution of their duties. In addition, nurses felt themselves to have been inadequately prepared for the task and expressed the need for continuous support and training. Some members of staff felt unappreciated because there was no follow-up to check whether they were recovering or were experiencing any difficulties.
	**Psychological effects of COVID-19 on nurses providing care** In the process of executing their duties, the nurses experienced considerable anxiety and fear associated with contracting the virus and transmitting it to their families. Witnessing patients’ experiences and distress was another source of anxiety and stress. Instances of stress were associated in particular with the loss of patients and having to communicate the news to the relatives or with patients’ experiencing discomfort such as respiratory distress. Participants who contracted COVID-19 felt traumatised by the experience and were of the view that the support system was either inadequate or lacking altogether.
**Outcomes**	Using the Donabedian model of care framework, it was possible to identify inefficient structures and processes. These had resulted in inefficiencies and/or gaps in the study setting that had negative psychological effects on healthcare workers. The healthcare work environment was overwhelming and emotionally draining.

*Source:* Please see the full reference list of the article Moyo, I., 2022, ‘Nurses’ experiences of providing care to suspected COVID-19 patients in a resource limited setting’, *Cogent Public Health* 9(1), 2058158. https://doi.org/10.1080/27707571.2022.2058158, for more information

PPE, personal protective equipment; COVID-19, coronavirus disease 2019; PCR, polymerase chain reaction test for COVID-19.

### Key study findings underpinning the development of the model

Donabedian’s structure–process–outcome framework (Donabedian 1988) was utilised to evaluate service provision in the context of COVID-19 from the perspective of healthcare workers at the frontline. The research findings reflect the gaps identified in the context of providing care. These gaps are discussed under each element of Donabedian’s model (structure, process and outcome) (Box 1).

The overall research findings showed the presence of healthcare delivery system inefficiencies and/or gaps in the study setting. The authors developed the model to close the identified gaps, guide the provision of psychosocial support for healthcare workers at the frontline as well as enhance health service delivery during the COVID-19 and other public health emergencies.

### Theoretical basis for the study

**FIGURE 2:**
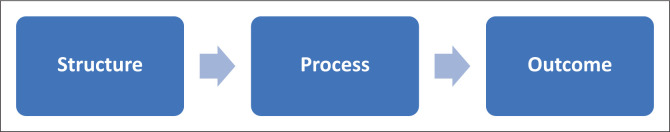
Donabedian’s structure–process–outcome framework.

The researchers chose Donabedian’s theory (Donabedian 1980a, 1980b) and the practice theory of Dickoff et al. (1968) as theoretical underpinnings for the study. The Donabedian framework (Figure 2) was found to be the most appropriate for this purpose because it encompasses all relevant aspects of the structure, process, and outcome of an organisation as well as the interrelationships between these elements (Donabedian 1988). The model is premised on the philosophical assumptions of Donabedian’s theory, and describes structure, process, and outcome measures as being interrelated and interdependent, with each being important to the overall environment in which care is provided (Donabedian 1980a, 1980b). In this instance, the focus was on delivery of health services during COVID-19 and the impact of this on the psychosocial well-being of nurses working at the frontline. Donabedian describes the structural measures of the model as referring to the environment and the resources necessary to enhance service provision, with these encompassing facilities, equipment, staff and financial resources. The process encompasses the techniques and practices utilised. Outcomes refer to the end results that have an effect on the recipients (nurses) in providing care. Process measures include delivery of care to patients and workflows. According to Donabedian, an effective structure is critical for the facilitation of an effective process, and ultimately, effective processes are in turn a prerequisite for high-quality outcomes (Botma & Labuschagne 2019; Donabedian 1985).

The findings of the phenomenological study were also conceptualised on the basis of Dickoff et al. (1968) practice theory elements: agents, recipients, context, process, dynamics, and outcome the theory poses six questions, which elucidate concepts and analyze the prescribed activities (study’s findings) as shown in Table 1.

**TABLE 1:** Elements of Dickoff et al. (1968).

Components of the theory	Application to the study
Context	Response of central hospitals city health department in Bulawayo to the outbreak of COVID-19
Agents	Hospital management Nurse managers
Recipients	Nurse practitioners working at the frontline during the COVID-19 pandemic
Process	COVID-19 response activities Provision of care to patients who contracted COVID-19 Conducting COVID-19 contact tracing activities Nurse practitioners receiving care as COVID-19 patients
Dynamics	Provision of counselling support Training: Motivation, mentoring and coaching
Outcome	Satisfaction of nurse practitioners Sustained support system Resilience of nurse practitioners

*Source*: Please see the full reference list of the article for more information

COVID-19, coronavirus disease 2019.

### The development of the model

To develop this model the following were used: Walker and Avant’s (2018) processes for theory development and Chinn and Kramer’s (2018) steps for model development.

#### Step one: Concept analysis

According to Walker and Avant (2018), concept analysis is a mechanism for identifying attributes essential for giving meaning to a particular concept. In this context, concept analysis formed the basis for the development of the psychosocial support model of caring for healthcare workers during a pandemic. In conducting concept analysis, the first step is concept selection (Walker & Avant 2018). In this case, the concept selected for this model was psychosocial support. The Donabedian theory has been viewed from a variety of lenses by several authors (Botma & Labuschagne 2019; Donabedian 1988). According to this Donabedian theory comprise three processes: structure, process and outcome. It is these components that laid the basis for the development of the model.

#### Step two: Synthesis and derivation

**FIGURE 3:**
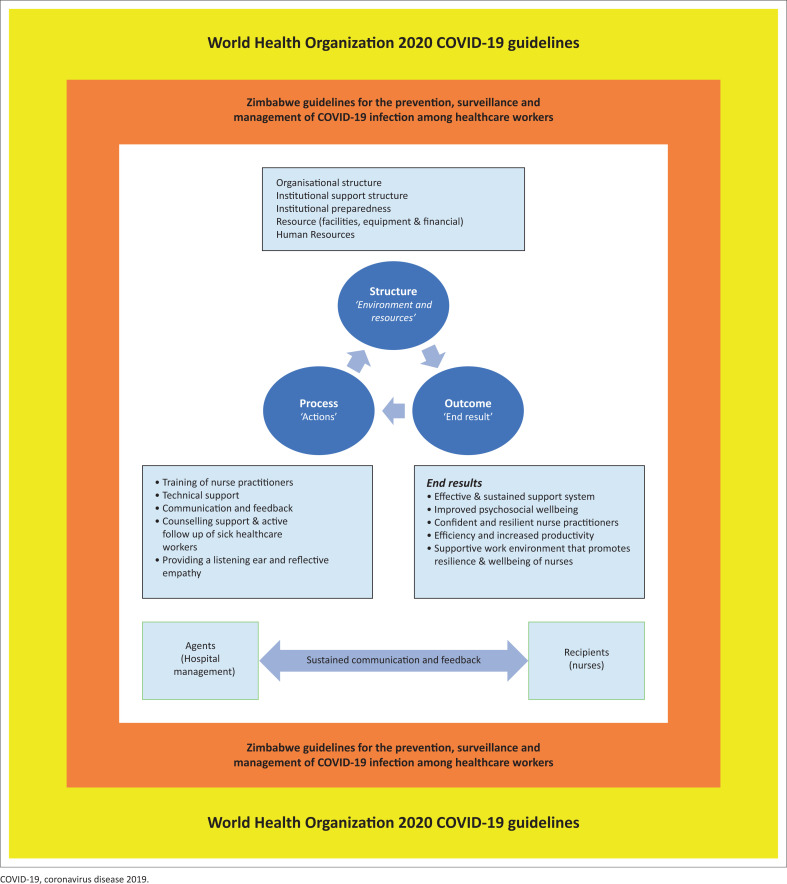
A psychosocial model of caring for healthcare workers during a pandemic.

**Synthesis:** Walker and Avant (2018), define synthesis as the generation of new ideas through examining data for novel insights. It can also be viewed as the development of statements about relationships by observing phenomena. Raw data mark the beginning of synthesis. The development of this model was informed by the research findings from phase 1 of the study, the reviewed literature and the Donabedian theory that underpinned the study. Phase 1 study explored the experiences of frontline healthcare workers who were either providers of care or sufferers of COVID-19. The study’s finding of phase 1 encompassed the following: a lack of institutional support structure, shortage of human and material resources, healthcare- and treatment-related costs. The study also found that COVID-19 took a psychological toll on these frontline healthcare workers. The results of concepts analysed using Donabedian’s theory were also utilised. The relationships among all these elements were part of the critical formulation of this model (see Figure 3).

**Derivation:** According to Walker and Avant (2018), theory derivation is a process of transposing or redefining a concept or theory from one context to another. This may be necessitated by the fact that existing theories are considered outdated and newer approaches are required. Walker and Avant (2018) posit that the aim of theory derivation is the development of strategies of explanation or predictions of poorly understood phenomenon or where current means to study them are lacking. Therefore, in this context theory derivation was utilised to link the research findings with the reviewed literature. In addition, the basic approaches of Walker and Avant (2018) and the Donabedian theory were adopted and adapted to develop the psychosocial support model of caring for healthcare workers during a pandemic.

### Evaluation and refinement of the model

The expert reviewers that contributed to the evaluation of the model are based in Zimbabwe and South Africa. The model was sent to five academics from South Africa and Zimbabwe for them to critically reflect on the model elements as outlined by Chinn and Kramer (2018). These were selected for the diversity of their skills and expertise in model development. They were asked to evaluate the model using Chin and Kramer’s (2018) evaluation guide, which included clarity, simplicity, generality, accessibility and importance of the model. As part of the evaluation process, two rounds of questionnaires were sent to the group of experts, as per the recommendations by Fletcher and Marchildon (2014). The two rounds enabled the expert reviewers to respond to and to revise their responses in view of the group members’ previous ones, until a consensus was reached (Wilkes 2015).

Feedback from experts was given to another group of healthcare workers who had experiences from the COVID-19 pandemic. The group that participated in the reflection of the model comprised healthcare workers from Bulawayo hospitals who were either sufferers or survivors from COVID-19 or carers of COVID-19 patients. They used their experience of COVID-19 in the evaluation of the model.

Following further adjustments, the model was presented and discussed in a meeting for nurses. The participants in this meeting confirmed that the model was clear, accessible, user friendly and could be utilised in other clinical settings particularly during pandemics.

### The description of the model

#### Purpose of the model

The major purpose of the model is to provide a frame of reference to guide healthcare service delivery. It also aims to initiate, develop and sustain a support structure that will contribute to an enabling work environment promoting efficiency and effectiveness in response to public health emergencies.

#### Assumptions

The model was underpinned by the philosophical assumptions of Donabedian’s framework (Donabedian 1988) and Dickoff et al. (1968) practice theory. From Donabedian’s perspective of structure, process and outcome, it is assumed that if all three elements are adequately attended to, an enabling healthcare environment prevails. This environment in its own way facilitates the provision of psychosocial support for healthcare workers:

- According to Donabedian, an established structure is a prerequisite for an effective process, and effective processes are a prerequisite for high-quality outcomes. In this context, if the healthcare providers are providing care in an enabling environment that is well resourced, this will enhance efficiency in providing care and related outcomes and ultimately improve the psychosocial well-being of the healthcare workers.
- It is also assumed that if a healthcare environment is supportive of its healthcare workers, this will enhance their productivity and efficiency.
- A healthcare worker is a bi-psychosocial being, therefore, building and providing an effective support system will enhance positive provider experience and resilience among healthcare workers.
- Nurses constitute the largest number of health providers who are at the frontline during public health emergencies. They, therefore, play a vital function in the response to the COVID-19 pandemic. This model advocates for a strong support system.
- A well-resourced healthcare environment (context) plays a critical role as a support structure for nurses involved in COVID-19 response activities and enhances performance as well as the psychosocial well-being of the nurse practitioner.

#### Elements of the model

The psychosocial model is described from the perspective of the elements of Donabedian’s framework (structure, process and outcome) and of Dickoff et al. (1968) practice theory elements (agents, recipients, context, process, dynamics and outcome) and within the national and international context of the COVID-19 pandemic.

### Context – COVID-19 response activities

According to Pfadenhauer et al. (2017), context is an overarching concept, comprising not only a physical location but also role interactions and relationships at multiple levels. Strauss and Corbin (1990) state that a context is characterised by a ‘specific set of properties pertaining to a phenomenon and a particular set of circumstances’ within which an action takes place. In the context of the study reported on in the present article, the context was the healthcare environment within which COVID-19 response activities took place. The context has an influence on both healthcare service delivery and the psychosocial well-being of players (nurse practitioners) involved (Montori et al. 2019). Figure 3 illustrates the context in which the model was developed. Below is a description of the context within which COVID-19 activities took place. The context is discussed at three levels: global, national and institutional.

### Global context

The global context (macro factors) is represented by the outer rectangle in Figure 3 and comprises the World Health Organization Guidelines on the Management of COVID-19 (WHO 2021).

### National context (Zimbabwe healthcare system)

The national context is regulated by the following legislative frameworks: the Zimbabwe Ministry of Health and Child Care COVID-19 Guidelines of 2020 and 2022, and the Guidelines for the Prevention, Surveillance and Management of COVID-19 infection among healthcare workers. The healthcare system in Zimbabwe is fragile, under-funded and under-resourced (Makoni 2020). While the financing model is both public- and donor-funded, the national budget still falls far below the 15% recommended by the Abuja Declaration (United Nations International Children’s Emergency Fund [UNICEF] 2021; Zimbabwe Ministry of Health and Child Care 2016).

### Healthcare institutions

As a context, the environment comprises central hospitals and city health facilities, with various healthcare providers (mostly nurses) involved in COVID-19 activities. These activities include caring for suspected or confirmed COVID-19 patients as well as conducting COVID-19 contact tracing. This context is regulated by the same legislative frameworks highlighted with regard to the national context, as well as institutional policies and protocols.

## Structure

The Donabedian framework covers all relevant aspects of an organisation’s structure, process, and outcome and their interrelations – hence its suitability for viewing or assessing an organisation, in this case, healthcare institutions (Donabedian 2005). For Donabedian, the structure is understood to be the attributes of material or human resources and organisational structure and includes the physical setting in which care takes place as well as the human resources, as represented by healthcare providers (Donabedian 1988, 2005).

### Material resources

Empirical findings from phase 1 of the study showed healthcare institutions as experiencing shortages of material resources such as PPE and other medical equipment. A shortage of human resources was a further obstacle. Evidence has demonstrated that the shortage of PPE and other medical equipment during a pandemic reduces the work efficiency of employees in that they experience high levels of anxiety, frustration, and insecurity (Cai et al. 2020; Watterson 2020). The inadequacies and gaps identified in the study affected the healthcare environment negatively, causing providers of care stress and anxiety. For COVID-19 response activities to be carried out efficiently and productively, it is recommended that the government and Ministry of Health and Child Care should undertake resource mobilisation so as to ensure a sustainable and adequate supply of medical equipment and resources to enhance productivity during public health emergencies of this nature.

### Human resources

Nurses play a critical role in the response to public health emergencies. It emerged from the study that nurses engaged in COVID-19 response activities were overwhelmed as a result of the increased workload, particularly during the first and second waves of COVID-19. For example, there were cases of nurses working for 12 h or more. A solution would be to recruit more staff to allow for flexibility in working shifts and prevent burnout.

### Institutional support

Empirical findings showed that the nurses engaged in COVID-19 activities felt that they were not appreciated by hospital management despite the daunting tasks they were required to carry out. Because of the movement restrictions introduced in response to the COVID-19 pandemic, nurses experienced difficulties with transport either to work or back to their homes. Meals were initially provided for the team involved in COVID-19 contact tracing activities, but these were later discontinued, a situation that had a negative impact on the nutritional status of the staff carrying out this work. Nurses who contracted COVID-19 struggled to pay medical bills, as they had to pay for laboratory investigations (COVID-19 polymerase chain reaction [PCR] testing) and X-rays at private facilities. The Ministry of Health and Child Care needs to develop innovative strategies for caring for the careers. In the given context, government support is required to provide a basic budget to cater for the welfare (transport, meals, medical bills) of healthcare workers at the frontline during a public health emergency.

## Processes

### Support structure

Arising from the pandemic were multiple stressors: fear of contracting the disease and transmitting it to family members, fear of loss of life, safety fears, supply shortages, increased workload, a sense of being overwhelmed and the long hours they were required to work. All these took a psychological toll on the nurses who participated in this study. Despite being drained both physically and emotionally, the providers of care nevertheless demonstrated resilience and commitment, and continued to execute their duties in a professional manner, even in the absence of a supportive structure. No supportive conversation such as counselling was provided. Ardebili et al. (2021) emphasised the pivotal role played by mental health support strategies such as counselling or other forms of supportive conversation during public health emergencies. To rectify this omission, institutions need to develop a structured supportive intervention strategy with dedicated staff for the provision of person-centered psychosocial support for healthcare providers either as providers of care or after contracting COVID-19. Most of the nurses who contracted COVID-19 received no follow-up during the time they were in isolation, the only contact being a call a day prior to the end of the isolation period to remind them about returning to work.

A support system structure, comprising a team whose mandate it is to provide psychosocial support to healthcare workers, conduct active (virtual) follow-up and offer counselling to staff and their significant others in isolation, is vital. Also critical to this is support to family members of a staff member who have contracted COVID-19 through the provision of information and counselling and feedback about their result as contacts. Such strategies were successfully implemented in the United Kingdom and United States (Greenberg et al. 2020; Jaklevic 2021), where time was allocated to providing counselling for distressed healthcare workers during COVID-19 by means of telehealth strategies. Based on lessons learnt from China, a structured psychological intervention programme is recommended. This will include creating awareness among healthcare workers of the need for psychosocial support during such emergencies, followed by the actual implementation of these services through telehealth (He et al. 2020). This will mean that from the outset designated crisis intervention groups need to be established to conduct virtual follow-up and offer supportive counselling for other members of staff who might have contracted COVID-19 or are providers of care at the frontline. The Ministry of Health and Child Care should set aside a budget to cater for the welfare of its healthcare workers during a crisis. Nurses who have contracted COVID-19, struggled to cover medical expenses and incurred out of pocket expenses should receive assistance from this budget.

### Communication

Communication between nurses working at the frontline and nurse managers was found to be ineffective. This was because the nurses did not have access to their managers for debriefing purposes or to speak about their experiences or problems they encountered during their participation in the COVID-19 response. Tomlin et al. (2020) emphasised the crucial role of communication during public health emergencies. Therefore, the authors call for an effective and sustained two-way communication structure between the nurses and management to keep staff informed or provide them with up-to-date information. If utilised, this proposed structure will ensure that healthcare workers are given an opportunity to express their fears and concerns via virtual platforms. An anonymous online platform needs to be created to allow healthcare workers working at the frontline to share insights into their experiences as they provide care in the context of COVID-19. According to Shanafelt et al. (2020), healthcare professionals ask five things of their organisation: hear me, protect me, prepare me, support me, and care for me. In addition, healthcare professionals would want the assurance that their opinions and expertise have been incorporated into the organisation’s emergency preparedness plans so as to improve the response to the pandemic.

## Main outcome of the research

The intention behind the model is to provide insights, support experiences and serve as a reference guide for Zimbabwe and other countries in the region in offering effective psychosocial support interventions and promoting the psychological well-being of healthcare workers during public health emergencies. It is envisaged that the use of this model will contribute to sustained psychosocial support for healthcare workers during COVID-19 and other public health emergencies. In addition, it is hoped that it will enhance efficiencies and productivity during unprecedented pandemics and build confident and resilient teams. It is also anticipated that use of the model will facilitate effective and sustained communication between nurses and their managers, which is vital during crisis such as pandemics. It is hoped that the model will also inspire health services managers and policymakers to formulate strategies for resource mobilisation and support for the development of structured supportive intervention strategies.

### Application of the psychosocial model

The model is intended as a contribution to the body of knowledge relating to public health emergencies and presents unique insights into the problems encountered in healthcare environments. In addition, the model proposes public health interventions to enhance support for healthcare workers involved in combating COVID-19 in a low-resource setting. The model can be utilised during pandemics and in any public healthcare crisis in other sub-Saharan countries or in other environment dealing with COVID-19. The implementation of this model will contribute towards establishing an enabling and supportive environment that facilitates efficiency. The current healthcare environment in Zimbabwe is fragile and under-resourced (Chingono 2019; Makoni 2020), a situation making it difficult to respond efficiently and effectively to the COVID-19 pandemic, and ultimately exerting a negative effect on the psychosocial well-being of nurses working at the frontline. The model also calls upon policymakers to ensure a strong supply chain system; this will require the government to mobilise adequate resources and to source funding for public health emergency response activities.

## Discussion

### Recommendations

Although the model was evaluated after its development, further piloting and monitoring of its implementation would be critical. The authors recommend its adoption, adaptation, and utilisation of this model in other low-resource settings particularly during epidemics and or pandemics.

### Limitations

The limitation of this study is that the model was not piloted as this was out of the scope of this study. The empirical data from healthcare providers was restricted to one province in the country; hence, findings report only the experiences in this province. However, the model would be useful in different healthcare settings in the sub-Saharan Africa.

## Conclusion

The fragile and under-resourced healthcare system has psychosocial implications to the well-being of healthcare workers. The challenges associated with the fragile and under-resourced healthcare system in the context of the COVID-19 pandemic resulted in psychosocial effects on the well-being of healthcare workers. The utilisation of this model is critical and facilitates the provision of an enabling and supportive environment that facilitates efficiency in response activities during pandemics.

This study provides a reference guide in the provision of psychosocial support for healthcare workers particularly during public health emergencies. There is paucity of evidence focusing on the well-being of healthcare workers during a crisis, hence the significance of this study.

## References

[CIT0001] Alase, A., 2017, ‘The interpretative phenomenological analysis (IPA): A guide to a good qualitative research approach’, *International Journal of Education and Literacy Studies* 5(2), 9–19. 10.7575/aiac.ijels.v.5n.2p.9

[CIT0002] Ardebili, M.E., Naserbakht, M., Bernstein, C., Alazmani-Noodeh, F., Hakimi, H. & Ranjbar, H., 2021, ‘Healthcare providers experience of working during the COVID-19 pandemic: A qualitative study’, *American Journal of Infection Control* 49(5), 547–554. 10.1016/j.ajic.2020.10.001 33031864 PMC7536124

[CIT0003] Botma, Y. & Labuschagne, M., 2019, ‘Application of the Donabedian quality assurance approach in developing an educational programme’, *Innovations in Education and Teaching International* 56(3), 363–372. 10.1080/14703297.2017.1378587

[CIT0004] Cai, H., Tu, B., Ma, J., Chen, L., Fu, L., Jiang, Y. et al., 2020, ‘Psychological impact and coping strategies of frontline medical staff in Hunan between January and March 2020 during the outbreak of coronavirus disease 2019 (COVID-19) in Hubei, China’, *Medical Science Monitor: International Medical Journal of Experimental and Clinical Research* 26, e924171. 10.12659/MSM.924171 32291383 PMC7177038

[CIT0005] Chingono, N., 2019, ‘Empty stomachs and unpaid salaries, Zimbabwe faces a bleak 2020 as economic crises deepens’, *CNN News*, viewed 13 August 2022, from https://www.cnn.com/2019/12/31/africa/zimbabwe-economic-crisis-intl/index.html.

[CIT0006] Chinn, P.L. & Kramer, M.K., 2018, *Knowledge development in nursing*, Elsevier Mosby, St. Louis, MO.

[CIT0007] Chinn, P.L., Kramer, M.K. & Sitzman, K., 2021, *Knowledge development in nursing e-book: Theory and process*, Elsevier Health Sciences, St Louis.

[CIT0008] De Raeve, P., Adams, E. & Xyrichis, A., 2021, ‘The impact of the COVID-19 pandemic on nurses in Europe: A critical discussion of policy failures and opportunities for future preparedness’, *International Journal of Nursing Studies Advances* 3, 100032. 10.1016/j.ijnsa.2021.100032 34079957 PMC8152362

[CIT0009] Dickoff, J., James, P. & Wiedenbach, E., 1968, ‘Theory in a practice discipline: Part I. Practice oriented theory’, *Nursing Research* 17(5), 415–434. 10.1097/00006199-196809000-00006 5186886

[CIT0010] Donabedian, A., 1980a, *Explorations in quality assessment and monitoring: The definition of quality and approaches to its assessment*. Health Administration Press, Ann Arbor.

[CIT0011] Donabedian, A., 1980b, *The definition of quality and approaches to its assessment*, Health Administration Press, New York.

[CIT0012] Donabedian, A., 1985, ‘The methods and findings of quality assessment and monitoring: An illustrated analysis’, *The Journal for Healthcare Quality (JHQ)* 7(3), 15.

[CIT0013] Donabedian, A., 1988, ‘The quality of care. How can it be assessed?’, *JAMA* 260(12), 1743–1748. 10.1001/jama.260.12.1743 3045356

[CIT0014] Donabedian, A., 2005, ‘Evaluating the quality of medical care’, *The Milbank Quarterly* 83(4), 691. 10.1111/j.1468-0009.2005.00397.x 16279964 PMC2690293

[CIT0015] Fletcher, A.J. & Marchildon, G.P., 2014, ‘Using the Delphi method for qualitative, participatory action research in health leadership’, *International Journal of Qualitative Methods* 13(1), 1–18. 10.1177/160940691401300101

[CIT0016] Greenberg, N., Docherty, M., Gnanapragasam, S. & Wessely, S., 2020, ‘Managing mental health challenges faced by healthcare workers during COVID-19 pandemic’, *BMJ* 368, m1211. 10.1136/bmj.m1211 32217624

[CIT0017] He, Z., Chen, J., Pan, K., Yue, Y., Cheung, T., Yuan, Y. et al., 2020, ‘The development of the “COVID-19 psychological resilience model” and its efficacy during the COVID-19 pandemic in China’, *International Journal of Biological Sciences* 16(15), 2828. 10.7150/ijbs.50127 PMC7545720 33061799

[CIT0018] Jaklevic, M.C., 2021, ‘Therapists donate their time to counsel distressed health care workers’, *JAMA* 325(5), 420–422. 10.1001/jama.2020.25689 33439225

[CIT0019] Liang, H.F., Wu, Y.C. & Wu, C.Y., 2021, ‘Nurses’ experiences of providing care during the COVID-19 pandemic in Taiwan: A qualitative study’, *International Journal of Mental Health Nursing* 30(6), 1684–1692. 10.1111/inm.12921 34369646 PMC8447461

[CIT0020] Makoni, M., 2020, ‘COVID-19 worsens Zimbabwe’s health crisis’, *The Lancet* 396(10249), 457. 10.1016/S0140-6736(20)31751-7 PMC7426087 32798478

[CIT0021] Montori, V.M., Hargraves, I., McNellis, R.J., Ganiats, T.G., Genevro, J., Miller, T. et al., 2019, ‘The care and learn model: A practice and research model for improving healthcare quality and outcomes’, *Journal of General Internal Medicine* 34(1), 154–158. 10.1007/s11606-018-4737-7 30430403 PMC6318165

[CIT0022] Moule, P., Aveyard, H. & Goodman, M., 2016, *Nursing research: An introduction*, Sage, Los Angeles.

[CIT0023] Moyo, I., Mgolozeli, S.E., Risenga, P.R., Mboweni, S.H., Tshivhase, L., Mudau, T.S. et al., 2021, ‘Experiences of nurse managers during the COVID-19 outbreak in a selected district hospital in Limpopo Province, South Africa’, *Healthcare* 10(1), 76. 10.3390/healthcare10010076 35052240 PMC8775488

[CIT0024] Moyo, I., 2022, ‘Nurses’ experiences of providing care to suspected COVID-19 patients in a resource limited setting’, *Cogent Public Health* 9(1), 2058158. 10.1080/27707571.2022.2058158

[CIT0025] Moyo, I., Mudzusi, A.H.M. & Haruzivishe, C., 2022, ‘Frontline healthcare workers’ experiences of providing care during the COVID-19 pandemic at a COVID-19 centre in Bulawayo, Zimbabwe: A phenomenological study’, *Curationis* 45(1), 1–11. 10.4102/curationis.v45i1.2292 PMC9257684 35792610

[CIT0026] Nie, X., Feng, K., Wang, S. & Li, Y., 2021, ‘Factors influencing public panic during the COVID-19 pandemic’, *Frontiers in Psychology* 12, 291. 10.3389/fpsyg.2021.576301 PMC7907154 33643123

[CIT0027] Pfadenhauer, L.M., Gerhardus, A., Mozygemba, K., Lysdahl, K.B., Booth, A., Hofmann, B. et al., 2017, ‘Making sense of complexity in context and implementation: The Context and Implementation of Complex Interventions (CICI) framework’, *Implementation Science* 12(1), 1–17. 10.1186/s13012-017-0552-5 28202031 PMC5312531

[CIT0028] Razu, S.R., Yasmin, T., Arif, T.B., Islam, M., Islam, S.M.S., Gesesew, H.A. et al., 2021, ‘Challenges faced by healthcare professionals during the COVID-19 pandemic: A qualitative inquiry from Bangladesh’, *Frontiers in Public Health* 9, 1024. 10.3389/fpubh.2021.647315 PMC8383315 34447734

[CIT0029] Shanafelt, T., Ripp, J. & Trockel, M., 2020, ‘Understanding and addressing sources of anxiety among health care professionals during the COVID-19 pandemic’, *JAMA* 323(21), 2133–2134. 10.1016/j.mayocp.2022.09.002 32259193

[CIT0030] Smith, J.A. & Osborn, M., 2015, ‘Interpretative phenomenological analysis as a useful methodology for research on the lived experience of pain’, *British Journal of Pain* 9(1), 41–42. 10.1177/2049463714541642 26516556 PMC4616994

[CIT0031] Strauss, A. & Corbin, J., 1990, *Basics of qualitative research*, Sage, Newbury Park.

[CIT0032] Tomlin, J., Dalgleish-Warburton, B. & Lamph, G., 2020, ‘Psychosocial support for healthcare workers during the COVID-19 pandemic’, *Frontiers in Psychology* 11, 1960. 10.3389/fpsyg.2020.01960 32849149 PMC7431467

[CIT0033] UNICEF, 2021, *Zimbabwe 2021 health budget brief*, viewed 17 July 2022, from https://www.unicef.org/zimbabwe/media/5176/file/2021%20Health%20Budget%20Brief%20-%20Final.pdf.

[CIT0034] Veugelers, R., Gaakeer, M.I., Patka, P. & Huijsman, R., 2020, ‘Improving design choices in Delphi studies in medicine: The case of an exemplary physician multi-round panel study with 100% response’, *BMC Medical Research Methodology* 20(1), 1–15. 10.1186/s12874-020-01029-4 PMC7294633 32539717

[CIT0035] Walker, L.O. & Avant, K.C., 2018, *Strategies for theory construction in nursing*, vol. 4, Pearson/Prentice Hall, Upper Saddle River, NJ.

[CIT0036] Watterson, A., 2020, ‘COVID-19 in the UK and occupational health and safety: Predictable not inevitable failures by government, and trade union and nongovernmental organization responses’, *New Solutions: A Journal of Environmental and Occupational Health Policy* 30(2), 86–94. 10.1177/1048291120929763 PMC7573676 32448036

[CIT0037] Wilkes, L., 2015, ‘Using the Delphi technique in nursing research’, *Nursing Standard* 29(39), 43. 10.7748/ns.29.39.43.e8804 26015141

[CIT0038] Xu, H., Stjernswärd, S. & Glasdam, S., 2021, ‘Psychosocial experiences of frontline nurses working in hospital-based settings during the COVID-19 pandemic – A qualitative systematic review’, *International Journal of Nursing Studies Advances* 3, 100037. 10.1016/j.ijnsa.2021.100037 34308373 PMC8285218

[CIT0039] World Health Organization (WHO), 2020, *Rational use of personal protective equipment for coronavirus disease (COVID-19): Interim guidance, 27 February 2020*, (No. WHO/2019-nCov/IPCPPE_use/2020.1). World Health Organization, Geneva.

[CIT0040] World Health Organization (WHO), 2021, *Living guidance for clinical management of COVID-19*, viewed 20 July 2022, from https://apps.who.int/iris/bitstream/handle/10665/349321/WHO-2019-nCoV-clinical-2021.2-eng.pdf.

[CIT0041] Zimbabwe Ministry of Health and Child Care, 2016, *Zimbabwe National Health Financing Policy Resourcing pathway to Universal Health Coverage*, Government Printers, Harare.

[CIT0042] Zimbabwe Ministry of Health and Child Care, 2020, *Zimbabwe guidelines for the management of COVID-19 Version April 2, 2020*, Government Printers, Harare.

[CIT0043] Zimbabwe Ministry of Health and Child Care, 2022, *Guidelines for the prevention, surveillance, and management of COVID-19 infection among health care workers guidelines in Zimbabwe*, Government Printers, Harare.

